# Early functional alterations in membrane properties and neuronal degeneration are hallmarks of progressive hearing loss in NOD mice

**DOI:** 10.1038/s41598-019-48376-x

**Published:** 2019-08-20

**Authors:** Jeong Han Lee, Seojin Park, Maria C. Perez-Flores, Wenying Wang, Hyo Jeong Kim, Leighton Izu, Michael Anne Gratton, Nipavan Chiamvimonvat, Ebenezer N. Yamoah

**Affiliations:** 1 University of Nevada, School of Medicine, Department of Physiology, Program in Communication & Sensory Sciences, Reno, NV 89557 USA; 2 Department of Internal Medicine, Division of Cardiovascular Medicine School of Medicine, University of California, Davis, CA 95616 USA; 3 Department of Pharmacology, University of California, Davis, CA 95616 USA; 4 Department of Otolaryngology, St Louis University, St Louis, MO USA; 5 Department of Veterans Affairs, Northern California Health Care System, Mather, 95655 CA USA

**Keywords:** Cochlea, Neurodegenerative diseases

## Abstract

Presbycusis or age-related hearing loss (ARHL) is the most common sensory deficit in the human population. A substantial component of the etiology stems from pathological changes in sensory and non-sensory cells in the cochlea. Using a non-obese diabetic (NOD) mouse model, we have characterized changes in both hair cells and spiral ganglion neurons that may be relevant for early signs of age-related hearing loss (ARHL). We demonstrate that hair cell loss is preceded by, or in parallel with altered primary auditory neuron functions, and latent neurite retraction at the hair cell-auditory neuron synapse. The results were observed first in afferent inner hair cell synapse of type I neurites, followed by type II neuronal cell-body degeneration. Reduced membrane excitability and loss of postsynaptic densities were some of the inaugural events before any outward manifestation of hair bundle disarray and hair cell loss. We have identified profound alterations in type I neuronal membrane properties, including a reduction in membrane input resistance, prolonged action potential latency, and a decrease in membrane excitability. The resting membrane potential of aging type I neurons in the NOD, ARHL model, was significantly hyperpolarized, and analyses of the underlying membrane conductance showed a significant increase in K^+^ currents. We propose that attempts to alleviate some forms of ARHL should include early targeted primary latent neural degeneration for effective positive outcomes.

## Introduction

Age-related hearing loss (ARHL) is the prevalent form of sensory deficit worldwide. The disease remains less understood, owing to the apparent late-onset phenotype and confounding factors, such as ototoxic drugs, noise trauma, and genetic pre-dispositions^1–3^. Although the frequency ranges in which mice (~1–90 kHz) and humans (~0.60–20 kHz) hear are distinct, similarities between the anatomy and physiology of the cochlea make the mouse a compelling animal model to study the mechanisms for ARHL in humans^1,4–6^. Previous studies of mutations linked to deafness in mice have provided important insights into human congenital deafness-associated genes^7–10^. Additionally, several inbred mouse strains exhibit progressive non-syndromic hearing loss that is expressed phenotypically at advanced ages, which mirrors ARHL in humans^11,12^. However, some of the missing mechanistic information for ARHL models is the detailed characterization of early events that occur in the cochlea.

Previous studies have described several ARHL mouse models. The *ahl* locus on chromosome 10 is the hub for the *cadherin* 23 (*Cdh23*) gene^12,13^, identified as a major contributor to ARHL in several mouse strains^14^. Mutations of two alleles, *Cdh23*^*ahl*^ and *ahl2*, are responsible for hair cell (HC) loss and an unidentified inner ear pathology, respectively^14–16^. These studies have provided a detailed characterization of the pathology of the cochlea in ARHL, but the functional neural mechanisms remain largely unknown. One of the prevailing views is that neuronal degeneration occurs secondary to HC loss^14^. Moreover, recent reports have implicated changes in synaptic^17^ and neuronal functions^18^ as primary sources for early hearing loss. Other studies have ascribed reduction in the endocochlear potential as a major factor in the etiology of hearing loss^14,19^. The best well-described features of ARHL in these models is basal-to-apical progressive HC loss and secondary neuronal loss^14^; which are also features shared by models of noise-induced hearing loss (NIHL)^20–23^. Recent reports have demonstrated that degeneration of afferent neurites may precede HC loss in NIHL models^17,24,25^, raising the possibility that there are early but “silent” neural mechanisms associated with NIHL that could be exploited to alleviate the long-term effects of the disease.

Previous studies have identified the non-obese diabetic (NOD) mouse as an ARHL model. The NOD mouse model exhibits multiple delayed symptoms such as immune deficits and metabolic defects, including symptoms of diabetes^26–28^. The NOD diseased phenotype is complicated by the fact that the onset of the disease is also gender-dependent, with symptoms affecting females by 10-weeks and males by 15-weeks of age^28,29^. Moreover, before the full-blown manifestation of the disease, changes in gene expression, and fluctuations in blood glucose levels have been reported^26,30^. It turns out that circadian rhythms may alter susceptibility to hearing loss^31^, suggesting the use of the NOD mouse model for long-term studies of hearing loss may be fraught with caveats. Here, we used the NOD mouse model to study mechanisms of early hearing loss.

We demonstrate that the NOD mouse model shows latent neuronal degeneration of afferent neuron-HC synapse before, and simultaneously with any outward manifestation of robust HC loss. Type II neurons were the first to degenerate. Moreover, we demonstrate that functionally, type I neurons undergo a step-wise reduction in membrane electrical properties with increased K^+^ current density leading to reduced excitability. There were significant alterations in membrane input resistance, resting membrane hyperpolarization, increased action potential (AP) thresholds, and latency in spiral ganglion neurons (SGNs) as early as 2 weeks postnatally at the onset of hearing. We propose that latent neuronal degeneration may represent one of the targets to alleviate some forms of ARHL.

## Results

### Progressive hearing loss in NOD mice

**Figure 1:**
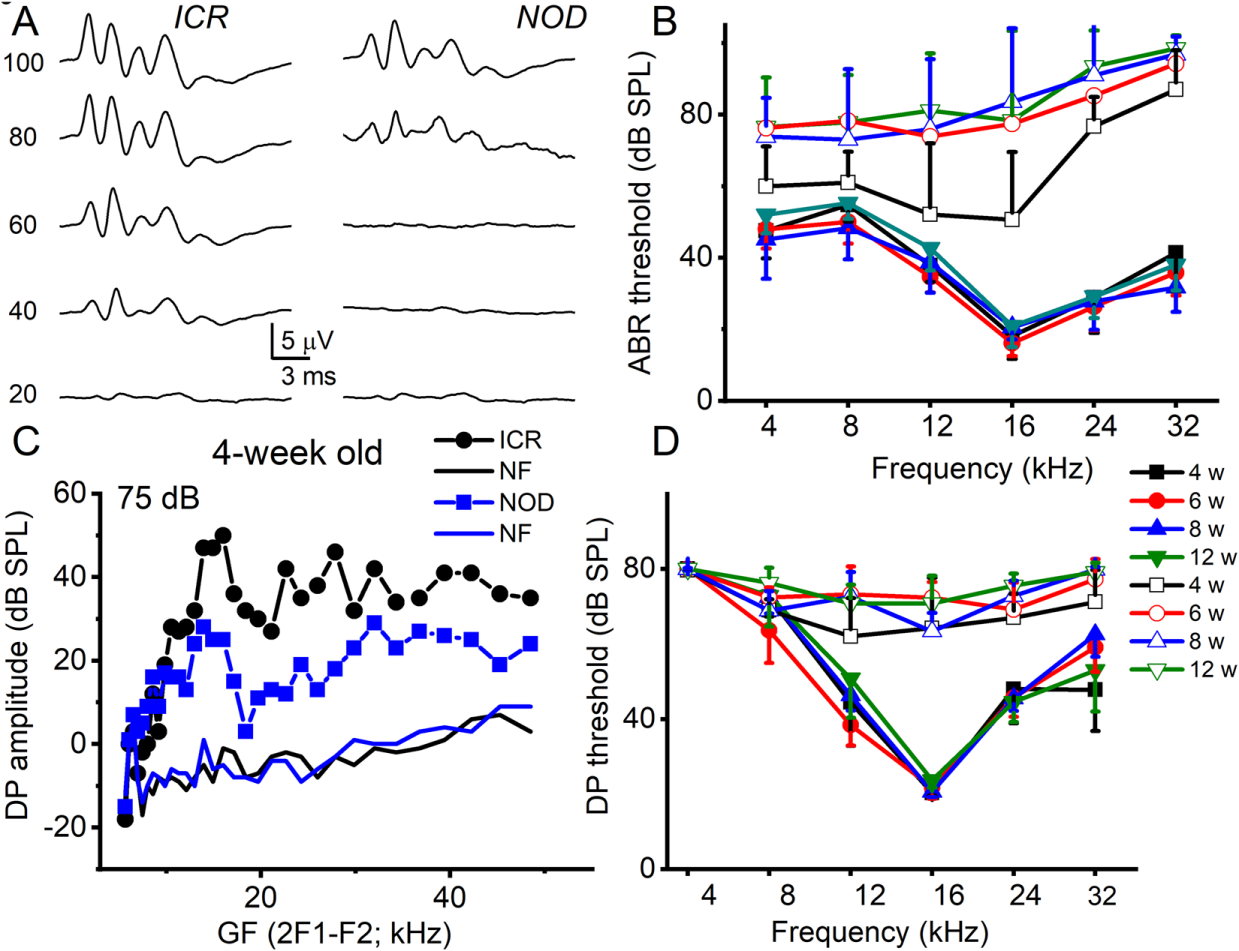
Progressive hearing loss in NOD mice. (**A**) Representative auditory brainstem response (ABR) waveforms from 4-week-old NOD mice (right panel) compare to age-matched ICR/HAJ (ICR) control mice (left panel). Clicks at different sound pressure levels (SPL; indicated) were used to invoke the waveforms. We used SPLs ranging between 10–100 dB at 10 dB intervals. (**B**) Plots of ABR threshold values from pure tone stimuli (4–32 kHz). Data were obtained from 11 mice from each group (4–12-week-old). (**C**) DPgrams for 4-week-old ICR control (in black) and NOD (in blue) mice at sound pressure level (SPL) 75 dB. The corresponding background (noise floor, NF) levels are plotted with continuous lines. (**D**) Average DPOAE threshold for NOD (*n* = 19) and their age-matched ICR control (4–12-week-old; *p* < *0.01*, *n* = 19) mice.

Using auditory brainstem response (ABR) and distortion product otoacoustic emissions (DPOAE) assessment, we examined the longitudinal progression of changes in hearing thresholds in 4–12-week old *ICR/vHaJ* and *NOD*.*CB17-Prkdc*^*scid*^*/J* mice, henceforth referred to as ICR control and NOD mice, respectively. We restricted the study from 2–12-week-old mice. The ICR control and NOD mice were age-matched. Equal numbers of males and females were used. Unless stated otherwise, when odd numbers of animals were used, females outnumbered males. Shown in Fig. 1A are exemplary averaged ABR traces recorded from 4-week-old ICR control and NOD mice. In stark contrast to the ICR control mice, the hearing thresholds for the NOD mice were significantly elevated across all frequencies (4–32 kHz) as early as four weeks (Fig. 1B; *p* < 0.05, see legend). The hearing thresholds for the ICR controls match well with other mouse stains^11^. By six weeks, the hearing threshold had increased and continued to increase in 12-weeks compared to their age-matched ICR controls. Consistently, the elevation of ABR hearing threshold progressed from high-to-low frequencies. We tested the outer HC (OHC) functions by assessing DPOAE. From the ABR data and previous studies^14^, the increased thresholds in distortion products suggest OHC loss and dysfunction (Fig. 1C,D). The loss of OHCs may contribute towards the increased ABR threshold. It is also conceivable that the auditory phenotype may consist of a mixture of congenital hearing loss with a component of ARHL.

### HC loss in NOD mice

**Figure 2:**
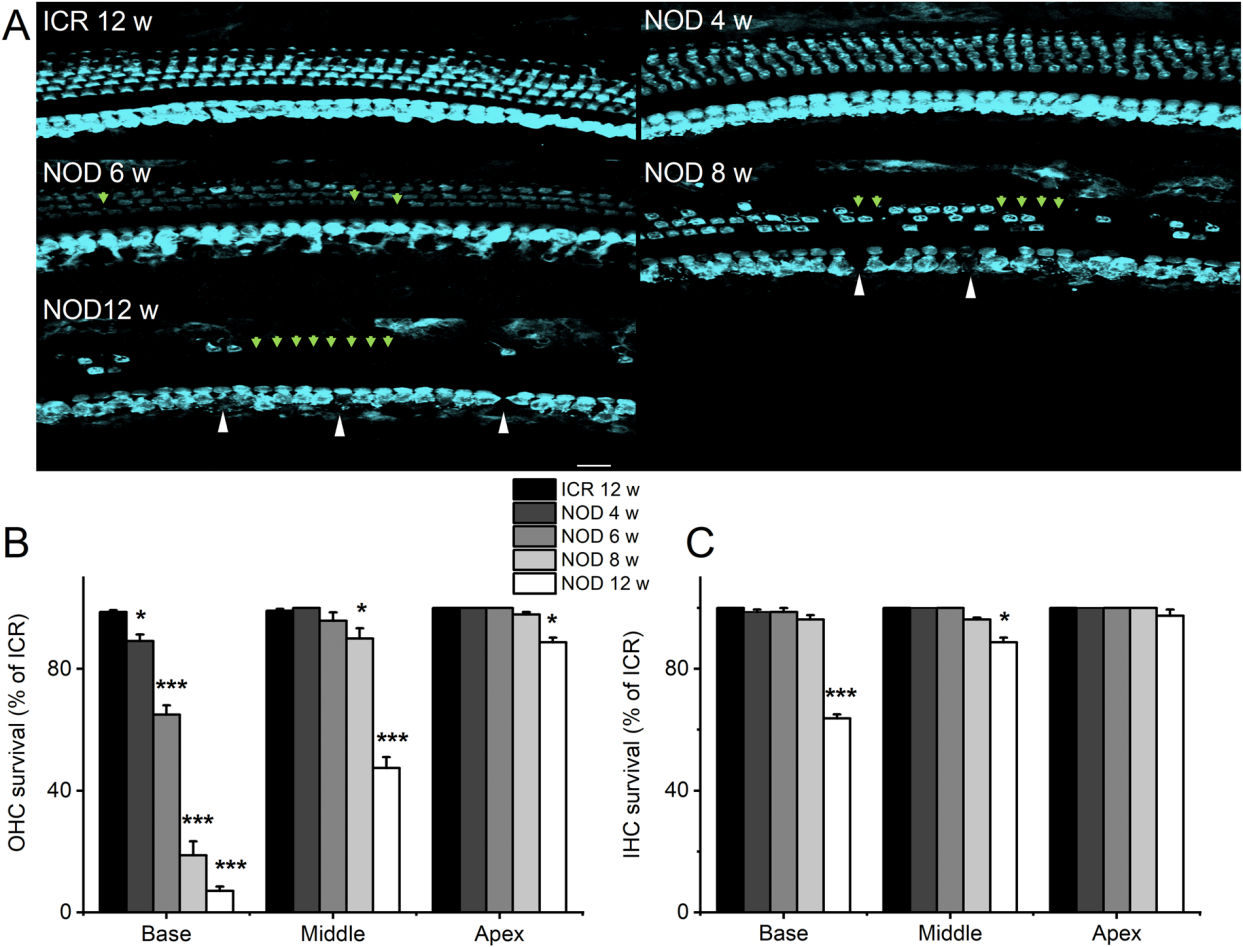
Hair cell loss in NOD mice. (**A**) Representative whole-mount preparations of the cochlear epithelium immunolabeled for anti-myosin 7 A (myo7a). The photomicrographs were obtained from the middle turn. Images from 12-week-old ICR control cochlea (left panel), showing inner hair cells (IHCs) and outer hair cells (OHCs). Images from 4–12-week-old cochlea indicated. White arrowheads indicate abnormal and loss of IHCs. Green arrowheads denote abnormal structure and missing OHCs in NOD mouse cochleae. (**B**,**C**) OHCs and IHCs were counted for quantification and shown as bar graphs. Error bars indicate, means ± s.d. *p < 0.05, **p < 0.01, ***p < 0.001. Scale bar = 15 µm.

Although earlier studies have shown that one of the cochlear defects in the NOD mice is strial degeneration and a decline in the endocochlear potential (EP), the loss of sensory cells precedes lateral cochlear wall dysfunction^14,19^. Therefore, we focused our attention on analyses of HC and SGN loss and the potential underlying cellular mechanisms. To ensure a thorough longitudinal study, we surveyed changes in HC numbers from the apex, middle, and basal segments of the cochlea in NOD mice in ages ranging from 2 to 12 weeks old. Figure 2 outlines the changes in HC numbers beginning from basal to apical aspects of the cochlea from 4–12-week-old NOD mice. By four weeks old, degeneration of cochlear OHCs had already begun at the base, progressing to the middle and apical regions. Subsequently, we observed significant degeneration of inner HC (IHCs) by 12 weeks. The summary data, depicted in Fig. 2B,C, were analyzed from 30 different cochlear samples for each age group and counted by five individuals in a blinded fashion.

Cadherin 23 forms part of structural proteins in hair bundle tip link^32,33^, and mutations associated with the protein result in hearing loss^34,35^. Indeed, the expression of the *Cdh23* ^*ARHL*^ allele is ascribed to ARHL^12,16^. Since OHCs at the basal aspects of the cochlea undergo early degeneration, we focused on changes in hair bundle morphology of apical OHCs. At the resolution of confocal microscopy, we did not observe any noticeable changes in the classical V-shape orientation of hair bundles of 2-week-old NOD mice (not shown). However, by 4-weeks, disruption of apical hair bundle was apparent, likely a preamble event towards HC degeneration. The disarray of hair bundles of apical OHCs continued, and by 12 weeks, IHC bundles and swelling of their basolateral membranes became visible (Fig. 2).

### Disruption and degeneration of type II SGNs and OHCs in NOD mice

**Figure 3:**
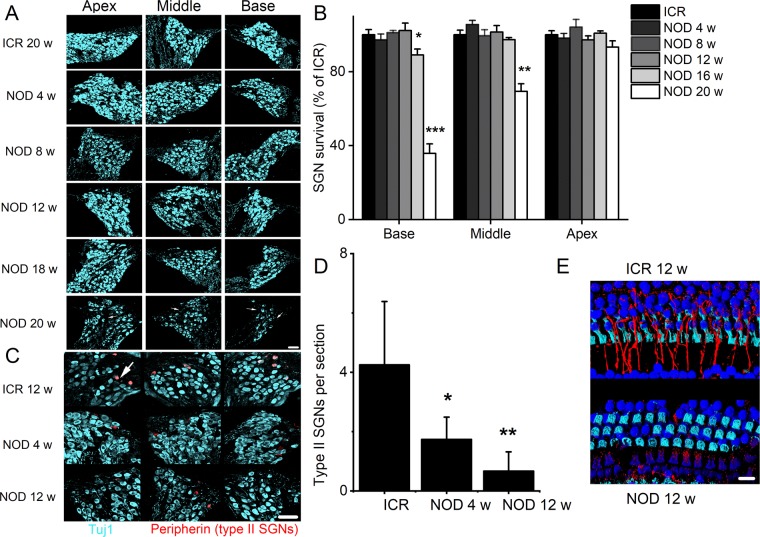
Assessment SGN cell bodies in ICR control and NOD mice, and early degeneration of type II SGNs. (**A**) Cryosections of the apical, middle and basal portion of the cochlea showing SGNs of 20-week-old ICR controls (upper panel). The lower panels illustrate the 4–20-weeks old NOD cochlear sections. SGNs were stained using anti-Tuj1, a neuronal marker (cyan). Arrows indicate the predicted sites of missing SGN soma. (**B**) Bar graph showing the mean numbers of SGNs that were counted for quantification by five individuals who were blinded to experimental ages of control and NOD cochleae. Error bars indicate, means ± s.d. *p < 0.05, **p < 0.01. Scale bar: 30 µm. (**C**) Cross-sections of SGNs were immunostained with anti-Tuj1 (cyan) for neurons and anti-peripherin (red) for type II neurons. The data were obtained from 12-week-old control mice and 4 and 12-week-old NOD mice. Arrow indicates type II neurons of SGN (in red). Scale bar = 30 µm. (**D**) Numbers of type II SGN were counted for quantification by 5-blinded individuals, and the averages were used to generate the bar graph. Error bars indicate, means ± s.d. *p < 0.05, **p < 0.01 (n = 41 sections from 5 different cochleae). Data were collected from samples imaged as a flat-mount, using confocal microscopy. (**E**) In control mice, peripherin-positive neurite projections from type II afferents. There is an obvious reduction in afferent innervation in 12-week-old NOD mice compared to controls. Scale bar = 10 µm.

Degeneration of SGN cell body is a shared feature of several models of ARHL, and invariably it has been described as a secondary event after HC loss^14,19^. We evaluated progressive changes in the number of spiral ganglion cell (SGC) bodies from the apex, middle, and base of the cochlea using a random sampling method (Fig. 3). The sampling was conducted by five individuals, who were blinded to the 20 different experimental preparations for each age group (Fig. 3A,B). The summary data suggested that cell bodies of auditory neurons of NOD mice remained intact until after 12 weeks when the number of neurons at the base and middle aspects of the cochlea decreased significantly (Fig. 3B). The spiral ganglion consists of ~95% type I and ~5% type II neurons^36,37^. The assessment made in Fig. 3A could not distinguish between the two cell types. Type II neurons have been identified as peripherin-positive cells^38–40^ (Fig. 3C–E). As it turns out, peripherin-positive cells in the NOD mice spiral ganglion undergo early and rapid degeneration starting at four weeks. Indeed, in 12-week-old mice, type II neurons were virtually absent as illustrated and summarized in the bar graph (Fig. 3C,D). As shown in Fig. 3E, by 12 weeks type II neurites have degenerated.

### Alteration of the normal juxtaposition of pre- and post-synaptic structures

**Figure 4:**
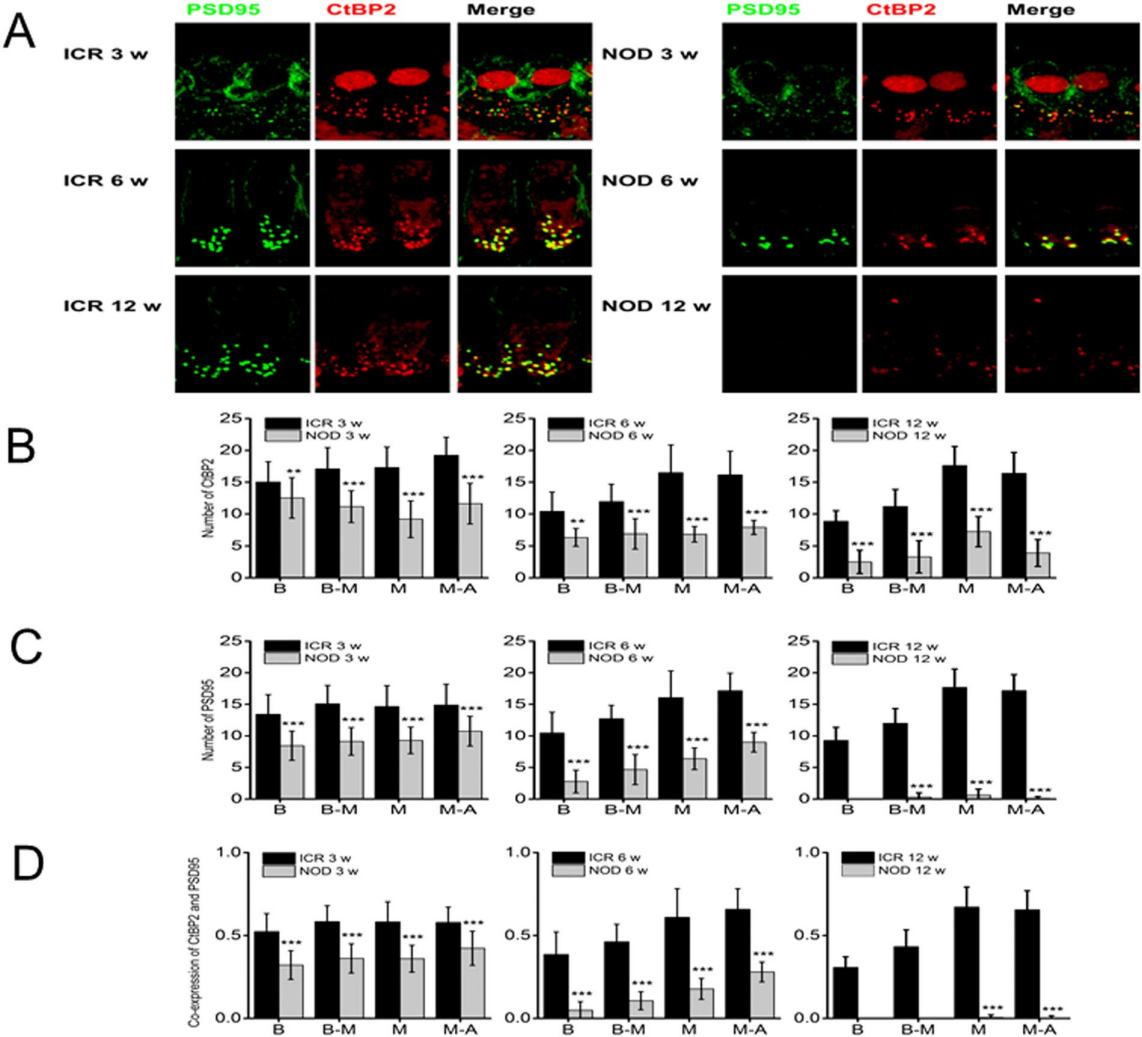
The number of presynaptic afferent synapses and postsynaptic densities per IHC in control and NOD mouse cochleae. (**A**) Confocal reconstruction of the organ of Corti with immune labeled ribbons (stained for CtBP2, red) and postsynaptic density 95 (PSD95, green). CtBP2 and PSD95 were counted using conditional probability as described in the methods section. (**B**–**D**) Here we show data, which were assessed by five individuals who were “blinded” by the experimental protocol, showing the numbers of CtBP2-positive (**B**) and PSD95-positive (**C**) structures. (**D**) The alignment of PSD95- and CtBP2- positive labeled structures as determined by the conditional probability algorithm (see methods), demonstrate overlap of pre- and post-synaptic markers. Error bars indicate, means ± s.d. (ANOVA F = 7.9, *p* = 0.01, Holm-Sidak < 0.05; n = 45 from 5 preparation for each age group. Scale bar = 5 µm.

Next, we evaluated potential changes in HC-SGN afferent synapses by comparing age-matched ICR controls with NOD mice from 3–12-week-old. Pre- and post-synaptic structures were labeled with antibodies against C-terminal binding protein 2 (CtBP2, red) and postsynaptic density protein 95 (PSD95, green), respectively. Type I and type II neuronal cell bodies showed significant degeneration only after 4–12 weeks. The results were in stark contrast to the substantial reduction in PSD95-labeling in 3-week-old NOD mice compared to their age-matched ICR controls (Fig. 4A). In keeping with the base-to-apex pattern of sensory cell degeneration, loss of PSD95-labeling adjacent to basal HCs was the first latent sign of failing sensory structures in the inner ear. Subsequently, there was a corresponding reduction in the number of CtBP2-labeling in a base-to-apex manner (Fig. 4B,C). We used the ratio of PSD95: CtBP2 labeling as an index of synaptic mismatch, and as illustrated in the bar graph in Fig. 4D, significant alteration of the normal juxtaposition of pre- and post-synaptic structures occurred before any sign of HC and SGN loss. Having demonstrated that afferent neuronal terminals were the first gross structures to undergo degenerative changes, we investigated whether other ultrastructural and functional changes preceded or occurred parallel with SGN cell body degeneration.

### Histological changes in auditory neurons

**Figure 5:**
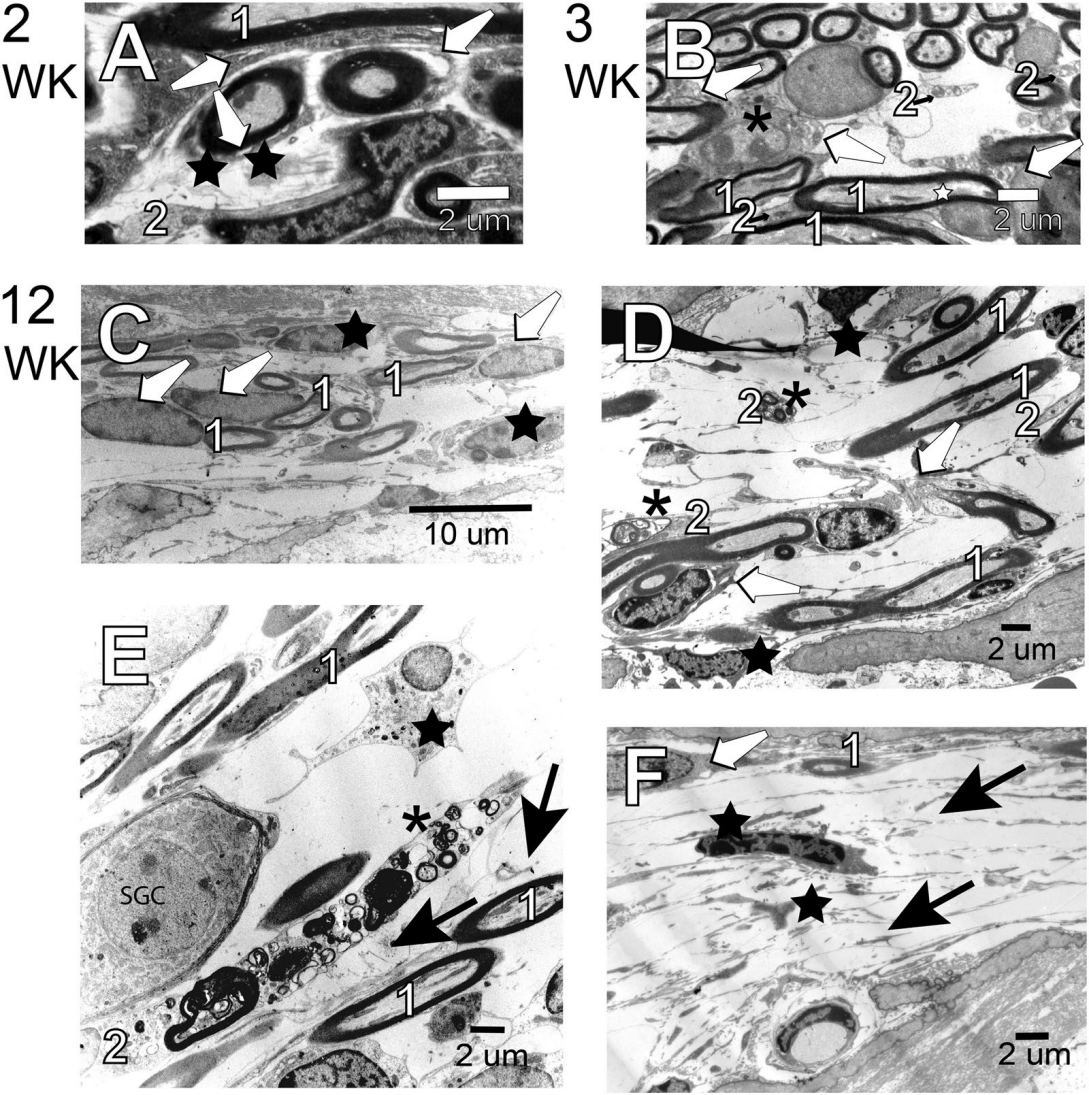
Primary auditory (8^th^) nerve fibers in the osseous spiral lamina. (**A**) Satellite cells in the basal turn show evidence of cytoplasmic condensation (white arrows). Macrophages (black stars) are present, and there is an increase in the amount of intercellular ground substance. Data from 2-week-old NOD cochlea. (**B**) At a similar basal location shows vacuoles and cristae diffluence in the cytosol of the satellite cell soma (white arrows as well condensation of nuclear chromatin (asterisk). Data from 3-week-old NOD cochlea. Some of the type I fibers (1) show cytosolic vacuoles (white star). Autophagosomes and evidence of mitochondrial architecture disruption are noted in some type II fibers (2). (**C–F**) Data from 12-week-old NOD cochlea. By 12 weeks of age, the degree of pathology is dependent upon the place located in the cochlear spiral. At lower magnification, **(C)** in the upper basal turn, loss of type I myelinated fibers (1) with few satellite cells (white arrows) is noted. Satellite cells (black arrows) contain autophagocytosed material while remnants of degenerated cells exist through the extracellular ground substance. No type II nerve fibers are noted. (**D,E**) At higher magnification, adjacent to the habenula perforata **(D)** as well as near the edge of Rosenthal’s canal **(E)**, the remaining type I fibers (1) show some normal mitochondria oriented with the length of the neuron, although the satellite cell (white arrows) for some neurons is degenerating. Most of the few type II nerve fibers (2) are undergoing autophagy with evidence of autophagosomes, auto-phagolysosomes, and dense-cored mitochondria. Macrophages (black star) with pseudo-podia and phagocytosed material are present. (**F**) Apart from a single type 1 nerve fiber (1) and satellite cell (white arrow), a section from the lower basal turn is devoid of normal cellular content. Two macrophages (black star), cellular debris and extracellular matrix now occupy this region. Scale bars. A, B, D–F: 2 μm, C: 10 μm.

We examined the histology of primary auditory neurons from 2–12-week-old cochleae. By 2-weeks in the NOD cochlea, several satellite cells in the basal turn show evidence of cytoplasmic condensation leading to separation of the cytosol from the myelin lamellae (Fig. 5A, white arrow). Macrophages, shown with black stars, had increased intercellular ground substance. At a similar basal location, we identified vacuoles and cristae diffluence in the cytosol of the satellite cell soma (white arrows as well as condensation of nuclear chromatin (Fig. 5B, asterisk). Some of the type I fibers show cytosolic vacuoles (white star).

**Figure 6:**
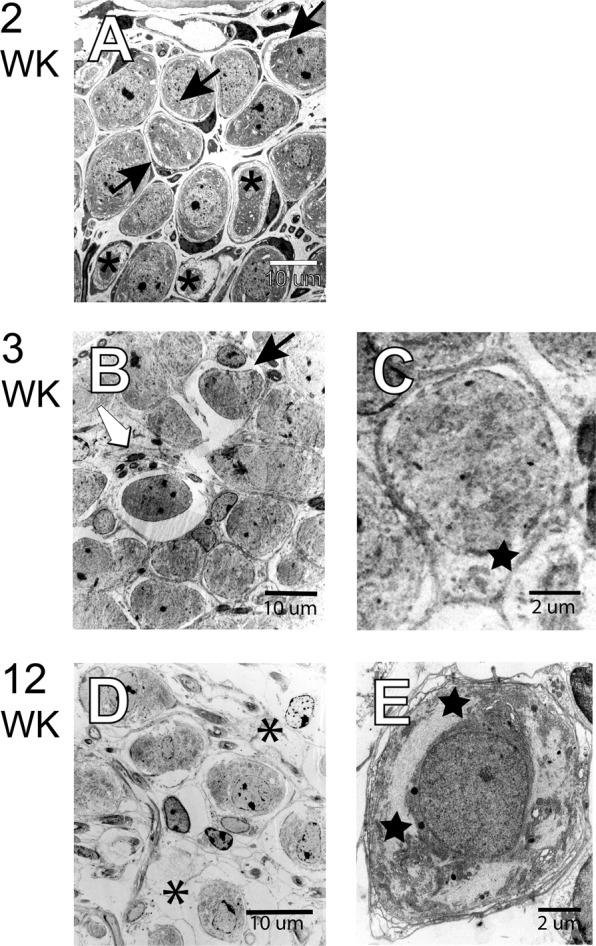
Spiral ganglion cells (SGCs) in upper basal Rosenthal’s canal. (**A**) Data obtained from 2-week-old NOD cochlea. Separation of the satellite cell and the SGC is evident (black arrow). Smaller sized SGCs (asterisk), presumably type II neuron have light-colored cytosol reflecting a loss of cellular organelles. (**B**,**C**) Data from the 3-week-old cochlea. At lower magnification, (**B**) separation of the satellite cell and SGC persist accompanied by the presence of dense-cored autophagosomes (white arrow). At higher magnification, (**C**) an SGC shows condensed cytosol with clumping of cellular organelles (star). (**D–F**) **1**2-week-old NOD cochlea. Satellite cells show a loss of cytosol and breakdown of the plasmalemma (asterisk). Cellular organelles tend to cluster along the plasmalemma and at opposite poles of the cell (stars). Scale bars, A, B, & D: 10 μm; C & E: 2 μm.

Additionally, autophagosomes and evidence of mitochondrial architecture disruption were noted in some type II fibers (Fig. 5B). By 12 weeks of age, the degree of pathology was dependent upon the place-location in the cochlear spiral. At low magnification, in the upper basal turn, loss of type I myelinated fibers with few satellite cells (white arrows) was noted (Fig. 5C). Some satellite cells shown with black arrows contained autophagocytosed material while remnants of degenerated cells existed through the extracellular ground substance. No type II nerve fibers were noted in the frame shown. At high magnification, adjacent to the habenula perforata (Fig. 5D), as well as near the edge of Rosenthal’s canal (Fig. 6E), the remaining type I fibers showed some normal long mitochondria oriented along the length of the neuron, although satellite cells (white arrows) for some neurons were degenerating. Most of the remaining type II nerve fibers were undergoing autophagy with evidence of autophagosomes, auto-phagolysosomes, and dense-cored mitochondria. Macrophages (black star) with pseudo-podia and phagocytosed material were identifiable (Fig. 5F). The histology of SGN cell bodies showed satellite cells separation, as evident in Fig. 6A (black arrow). Smaller sized SGCs (asterisk), presumably type II neurons, have light-colored cytosol reflecting a loss of cellular organelles. In 3-week-old NOD cochlear, at low magnification (Fig. 6B), separation of the satellite cell and SGC persisted and was accompanied by the presence of dense-cored autophagosomes (white arrow). At higher magnification (Fig. 6C), an SGC show condensed cytosol with clumping of cellular organelles (star). Finally, in 12-week-old NOD cochlea satellite cells show a loss of cytosol and breakdown of the plasmalemma (asterisk). Cellular organelles tended to cluster along the plasmalemma and at opposite poles of the cell (Fig. 6D–F, stars).

### Changes in membrane input resistance and membrane properties of SGNs in the NOD model

**Figure 7:**
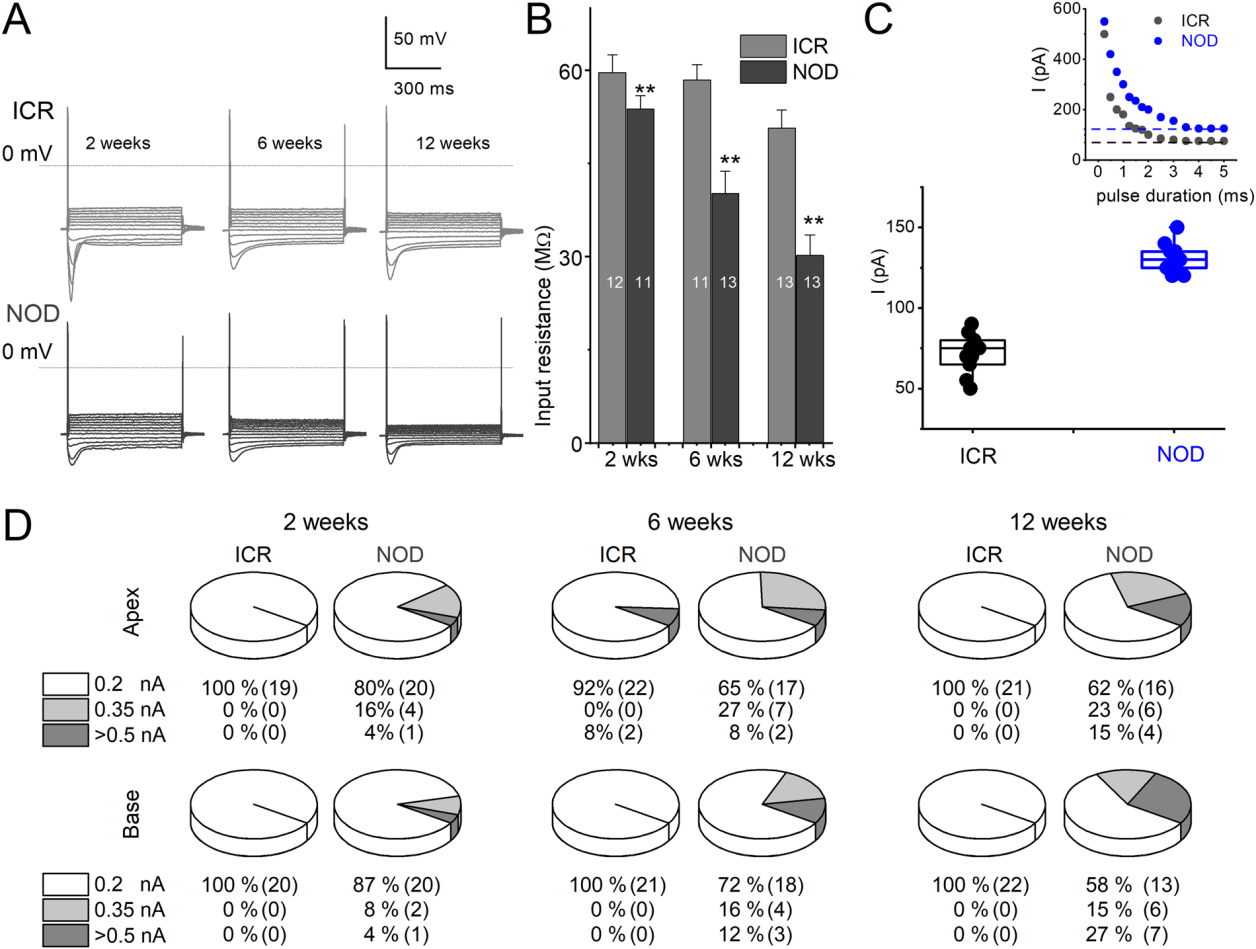
Changes in membrane input resistance of spiral ganglion neurons (SGNs) in the ARHL model. (**A**) Current-clamp protocol was used to examine the membrane input resistance with multiple current (I) steps (~600-ms in duration) between −0.2 and +0.35 nA, ΔI = 0.05 nA. (**A**) Family of traces obtained from ICR control (top traces) and NOD SGNs (bottom traces). Data were obtained from SGNs from 2-, 6- and 12-week-old cochleae. (**B**) Summary data of the input resistance, which was measured from changes in membrane potentials (V) as a function of injected currents (I). Bar graph depicts the reduction of input resistance in NOD mice compared to their age-matched controls. Number of SGNs at different age groups are indicated (*p* < 0.01). (**C**) Box plot of the mean rheobase current for 2 (solid symbols) and 12-(open symbols) week-old SGNs in ICR (in black symbols) and NOD (in blue symbols) mice. The inset is an example of a plot used to determine the mean rheobase current. (**D**) The pie chart shows the percentage of SGNs that elicited action potentials in response to different current injection. Note that SGNs from NOD mice were less responsive to membrane voltage changes upon current injection. ICR control group SGNs generated action potentials using ~0.2 nA current injection (n = 125) except for two neurons from the 6-week-old cochlea, at the apical region, which required more than 0.5-nA current injection.

To examine the baseline membrane conductance of SGNs, we determined the membrane input resistances in 2, 6, and 12-week-old neurons isolated from ICR control and NOD mice (Fig. 7). Injection of negative current in 2-week-old ICR control neurons exhibited characteristic membrane ‘jitters,’ which was reduced profoundly in the age-matched NOD neurons (Fig. 7A,B). The input resistance of neurons from NOD mice was reduced in 2-week-old SGNs compared to ICR control neurons and underwent progressive decline (Fig. 7B). To examine the extent of excitability of SGNs, we varied the magnitude and duration of injected current plotted current as a function of stimulus duration to determine the rheobase current for 2-week and 12-week old SGNs isolated from the apical cochlea (Fig. 7C). Spiral ganglion neurons from ICR controls were more excitable than the age-matched NOD mice. A survey of the proportion of neurons, which required more than 0.2-nA to evoke action potentials, increased longitudinally, and spatially along the cochlear axis (Fig. 7D). The data reflects the generalized reduction in membrane excitability in the NOD mouse neurons (Table 1).

**Table 1:** Summary of action potential properties of 2- and 12-week old ICR and NOD SGNs. *p < 0.05; ** < *0.01*. RMP = resting membrane potential.

	2-week				12-week			
	apex		base		apex		base	
	ICR	NOD	ICR	NOD	ICR	NOD	ICR	NOD
RMP (mV)	−58.7 ± 3.4 (19)	−61.8 ± 3.0 (25)**	−57.9 ± 2.5 (20)	−61.3 ± 2.9 (23)**	−62.8 ± 4.1 (21)	−66.1 ± 2.7(26)**	−61.3 ± 3.1 (22)	−64.2 ± 2.9 (26)*
threshold (mV)	−36.8 ± 3.1 (19)	−40.4 ± 3.5 (24)**	−36.3 ± 2.4 (20)	−39.0 ± 33 (22)*	−41.7 ± 3.2 (21)	−44.7 ± 4.2 (22)**	−40.2 ± 3.5 (22)	−43.4 ± 3.5 (19)*
latency (ms)	2.0 ± 0.1 (19)	3.0 ± 0.3 (20)*	2.0 ± 0.2 (20)	2.3 ± 0.2 (21)	2.3 ± 0.3 (21)	3.0 ± 0.3 (16)*	2.1 ± 0.2 (22)	2.7 ± 0.2 (15)*
amplitude (mV)	75.4 ± 7.2 (19)	69.5 ± 6.5 (20)**	82.8 ± 7.4 (21)	71.1 ± 8.8 (21)**	87.0 ± 9.1 (20)	80.9 ± 9.0 (20)*	80.7 ± 10.1	73.7 ± 8.4 (16)**

The number of neurons is indicated in brackets ().

**Figure 8:**
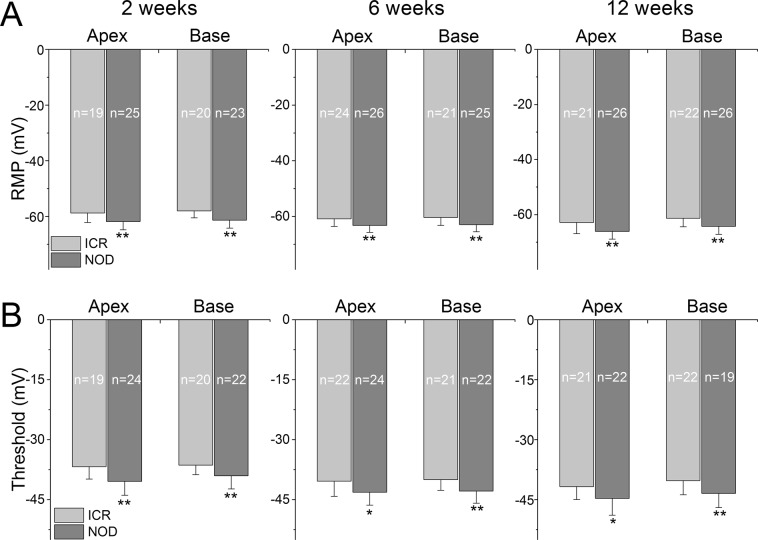
Changes of membrane properties of ICR *versus* NOD SGNs at post-hearing stages in 2-, 6- and 12-week-old. (**A**) The resting membrane potential (RMP) of NOD SGNs was hyperpolarized relative to ICR SGNs (**p < 0.01; n = as indicated, e.g. mean + std; 2-week old; Apex = −58.7 ± 3.4 (n = 19), and −61.8 ± 3.0 (n = 25): Base = −57.9 ± 2.5 (n = 20), and −61.3 ± 2.9 (n = 23); 6-week old; Apex = −60.8 ± 2.8 (n = 24), and −63.1 ± 2.5 (n = 26): Base = −60.3 ± 2.9 (n = 21), and −62.9 ± 2.6 (n = 25); 12-week old; Apex = −62.8 ± 4.1 (n = 21), and −66.1 ± 2.7 (n = 26): Base = −61.3 ± 3.1 (n = 22), and −64.2 ± 2.9 (n = 26). (**B**) Changes in the threshold potential of NOD neurons compared to control neurons are summarized (numbers of SGNs tested, indicated (*p < 0.05; **p < 0.01; 2-week old; A*p*ex = −36.8 ± 3.1 (n = 19), and −40.4 ± 3.5 (n = 24): Base = −36.3 ± 2.4 (n = 20), and −39.0 ± 3.3 (n = 22); 6-week old; Apex = −40.4 ± 3.8 (n = 22), and −43.1 ± 3.2 (n = 24): Base = −39.9 ± 2.7 (n = 21), and −42.8 ± 3.0 (n = 22); 12-week old; Apex = −41.7 ± 3.2 (n = 21), and −44.7 ± 4.2 (n = 22): Base = −40.2 ± 3.5 (n = 22), and −43.4 ± 3.5 (n = 19)).

**Figure 9:**
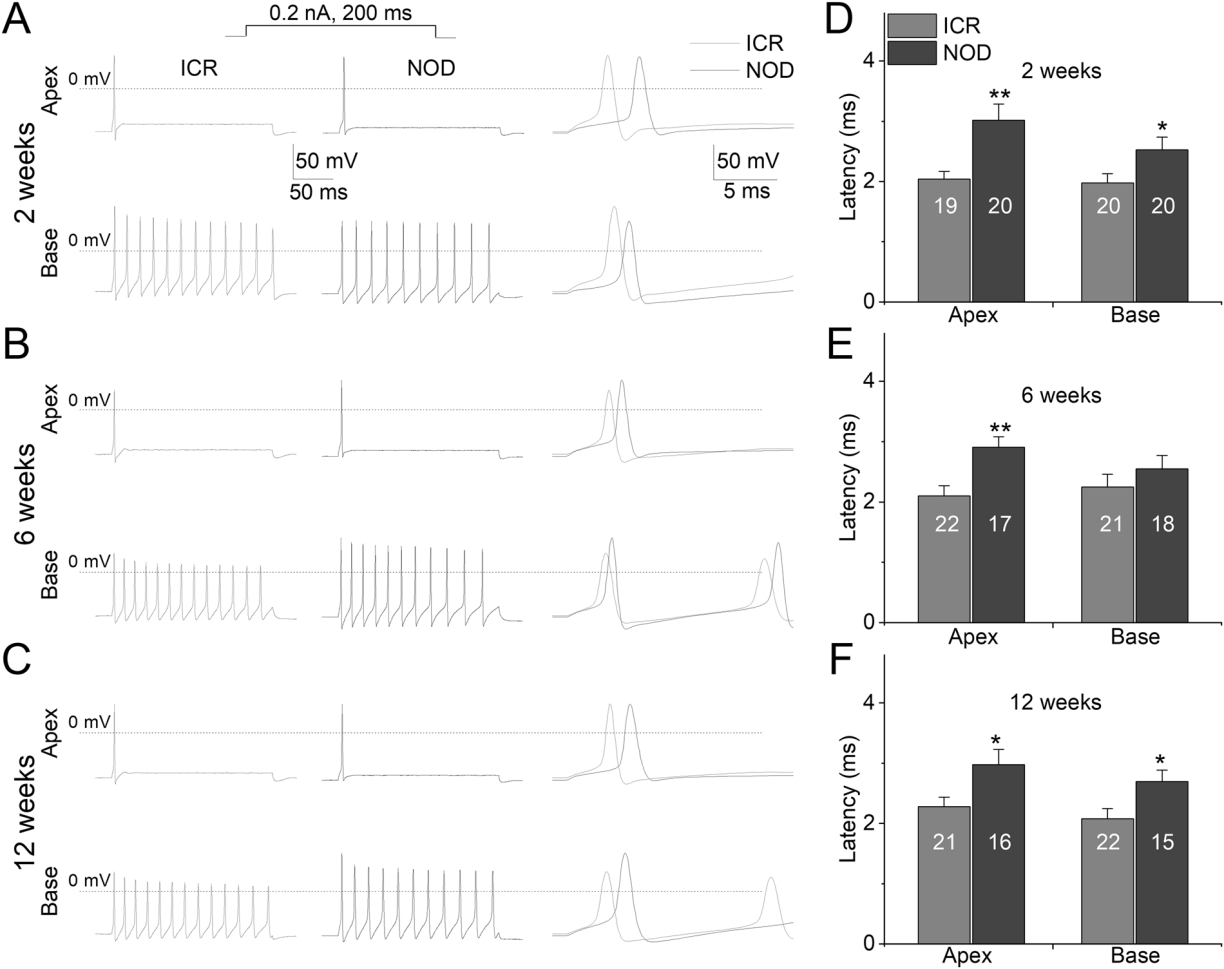
Changes in action potential properties in SGNs in NOD SGNs. (**A–C**) Representative traces of action potentials recorded from ICR (left traces) and NOD (middle traces) SGNs at different stages, 2-, 6-, and 12-week-old. Action potentials at the right panels are superimposed for comparison. (**D–F**) Mean spike latency for NOD and their age-matched control show a significant prolongation relative to ICR control SGNs. (* p < 0.05, ** p < 0.01; n = indicated, e.g. mean ± std; 2-week old; Apex = 2.04 ± 0.13 (n = 19), and 3.02 ± 0.27 (n = 20): Base = 1.98 ± 0.15 (n = 20), and 2.53 ± 0.21 (n = 20): 6-week old; Apex = 2.10 ± 0.17 (n = 22), and 2.90 ± 0.17 (n = 17): Base = 2.25 ± 0.21 (n = 21), and 2.55 ± 0.22 (n = 18): 12-week old; Apex = 2.28 ± 0.16 (n = 21), and 2.98 ± 0.25 (n = 16): Base = 2.08 ± 0.17 (n = 22), and 2.70 ± 0.19 (n = 15).

Additionally, the resting membrane potential (RMP) of the NOD SGNs remained relatively hyperpolarized, and the activation threshold was elevated compared to their ICR control neurons (Fig. 8). This trend remained intact during 2–12 weeks (see Table 1). Since there has been a reported case of gender differences in phenotype in the NOD mouse model^28,29^, we grouped the data into males and females. We did not observe statistical differences between male and female NOD mice (data not shown). Consistent with the excitability differences between the ICR control and NOD neurons, action potential latencies in NOD neurons were significantly prolonged compared with age-matched control mice (Fig. 9).

### Profound reduction in membrane excitability of SGNs from the NOD mice

**Figure 10:**
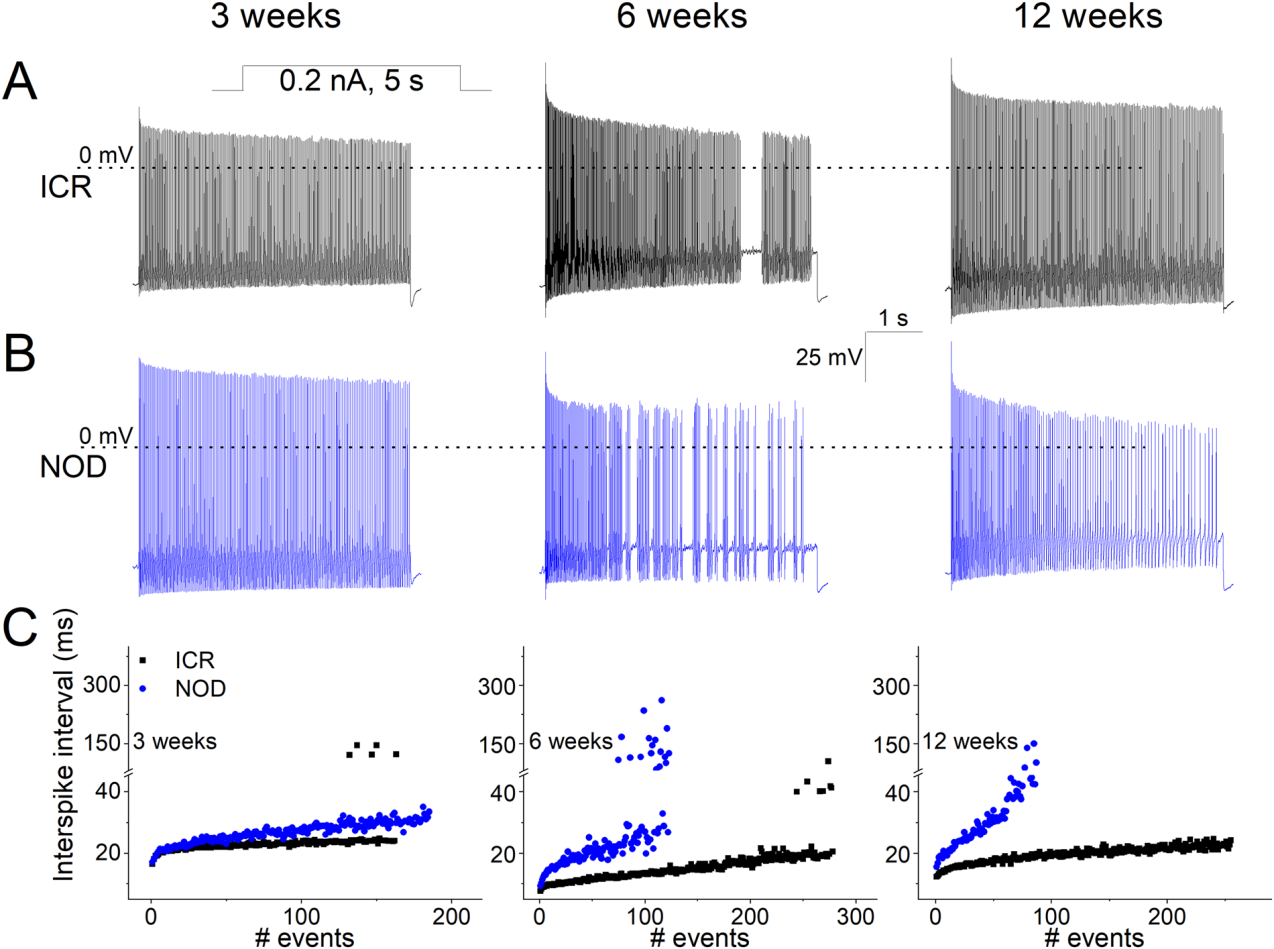
Alteration in the firing of SGNs with stimulus-evoked burst activity in ICR controls and NOD mice. (**A**) Action potentials were recorded from SGNs in ICR mice by injecting a 0.2-nA current for the 5-s duration. Shown are examples of recordings obtained from 2-, 6- and 12-week-old SGNs from ICR control mice (in black). (**B**) Similar protocol, as shown in (**A)** was used to invoke spike trains in NOD mice from 2-, 6- and 12-week-old SGNs (in blue). (**C**) The plot of inter-spike intervals reveals reduced excitability in NOD SGNs. The inter-spike intervals between controls and NOD mice remain essentially unchanged in 2-week-old SGNs.

**Figure 11:**
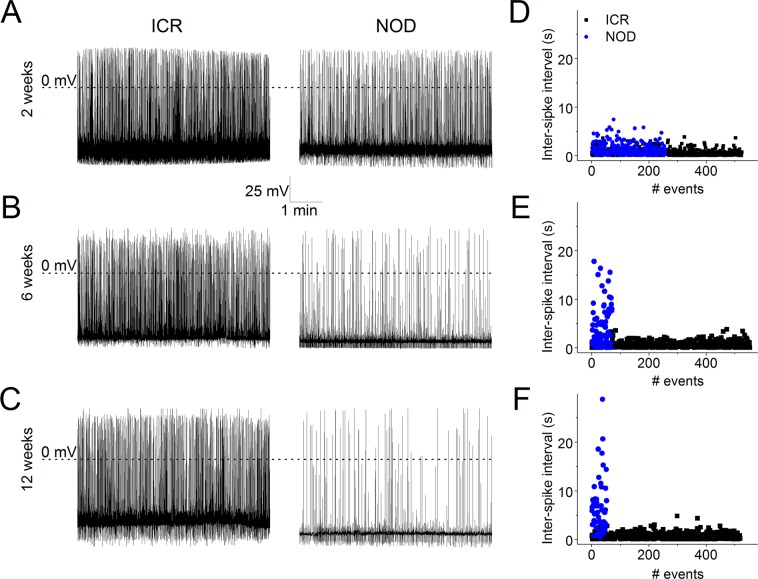
Assessment of SGNs with spontaneous activity in ICR controls and NOD mice. A subset of post-hearing SGNs remains spontaneously active. Shown are examples of such neurons in ICR and NOD SGNs in 2- (**A**), 6- (**B**), and 12-week-old (**C**) mice. (**D–F**) Inter-spike intervals plotted as a function of events. There were no discernible differences between the inter-spike intervals of control and NOD SGNs in 2-week-old. However, the number of events was reduced in the NOD SGNs. In contrast, by 6–12-weeks, there were stark reductions in the inter-spike intervals and the number of events in the NOD compared to control SGNs.

Post-hearing SGNs are heterogeneous in their stimulus responsiveness and whether they are spontaneously active^41–43^. The NOD and their ICR control neurons retained their mixed stimulus responsiveness ranging from fast, slowly adapting, and spontaneously active properties. Moreover, there were substantial differences in the rate of firings, as reflected in the inter-spike intervals of action potentials in slowly adapting neurons between the ICR controls and the NOD mice (Fig. 10). After six weeks old, the varied inter-spike intervals between the ICR and NOD were more apparent in slowly adapting neurons, and the spontaneously active group (Figs 10 and 11). These results consistently demonstrated the profound reduction in membrane excitability of SGNs from the NOD mice relative to their age-matched ICR controls.

### Enhanced whole-cell K^+^ current in SGNs from NOD compared to ICR mice

**Figure 12:**
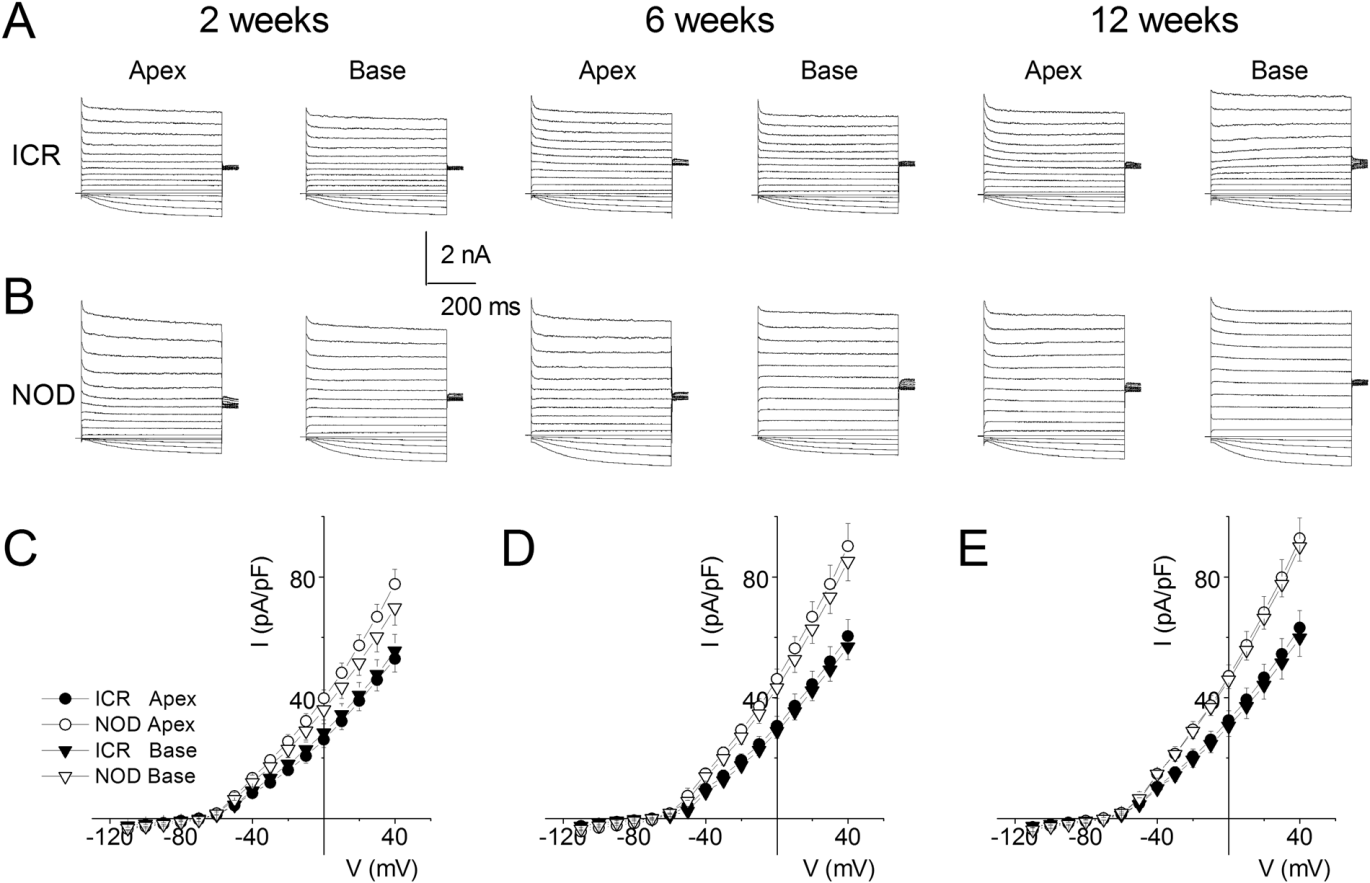
Enhanced whole-cell K^+^ current in post-hearing NOD compared to ICR control SGNs. (**A**) Voltage clamp recordings of apical and basal SGNs of 2-, 6-, and 12-week-old ICR mice. SGNs were held at −70 mV and stepped from −110 to +40 mV using 10-mV increments. (**B**) Similar recordings from NOD SGNs, using the same conditions. (**C–E**) Summary data of the current density-voltage (I/V) relationship showing that there was a significant increase in the current density of NOD SGNs in 2-, 6- and 12-week-old neurons (*p < 0.05; n = 17 at each location and age group).

We then determined the underlying conductance/s responsible for the hyperpolarized membrane potential and reduced membrane excitability of SGNs in NOD mice. Because of significant membrane hyperpolarization in the NOD neurons, we targeted outward K^+^ currents. In a similar fashion as described previously^41,43^, inward currents were blocked to evaluate the magnitude of the whole-cell K^+^ currents. We identified stark contrast between whole-cell outward K^+^ current amplitudes in SGNs of the NOD mice and their age-matched ICR controls beginning at two weeks of age (Fig. 12). The magnitude of the outward K^+^ current in the NOD neurons continued to increase relative to currents in control neurons for the duration of time studied (2–12 weeks). The precise identity of the K^+^ channel subtype/s that are altered in the aging SGNs would require future studies that are beyond the scope of the present report.

## Discussion

The findings from this report demonstrate that phenotypic manifestation of ARHL can be gleaned from ABR and DPOAE assessment by four weeks of age in NOD mice (Fig. 1). Compared to their age-matched controls, degeneration of postsynaptic densities and loss of synaptic ribbons begin as a latent phenomenon, serving as a preamble towards hearing loss as early as two weeks, as seen in changes in the ultrastructure of SGCs (Figs 3, 5 and 6). Reduction in membrane excitability of SGNs represented as a decrease in membrane input resistance, increased resting membrane hyperpolarization, AP activation threshold, and prolonged latency are hidden prefaces of ARHL (Figs 7–9). Indeed, a substantial increase in outward K^+^ current was determined in NOD SGNs relative to control neurons before morphological changes in HCs (Fig. 12). We attribute changes in membrane properties associated with ARHL to enhanced outward K^+^ currents. Most surprisingly, HC loss appeared secondary or in parallel with the initial afferent synaptic terminal withdrawal^14^ (Figs 2, 4) Cell bodies of peripherin-positive type II neurons degenerate ahead of type I neurons (Fig. 3). These discoveries have revealed several silent overtures before the outward expression of hearing the loss in the NOD ARHL model, and if found to be a shared property of other ARHL animal models may represent important targets for disease rescue, using growth factors known to alter SGN functions^44^.

### Significant alteration of the normal juxtaposition of pre- and post-synaptic structures

Previous studies have shown that NIHL and ARHL share common traits^45–47^ and that early noise exposure can exacerbate the progression of ARHL^24,25,48^. Because ARHL is implicitly a slow process manifesting as a chronic phenotype, it has been difficult to identify or establish whether there is an acute intrinsic event that initiates the progression of hearing loss. Morphological studies of IHC-afferent neuron synapses have demonstrated acute noise- and excitotoxic-induced nerve terminal swellings that may be reversible in some models^49–52^. Nonetheless, in cases where synaptic morphological changes are irreversible, anatomical alterations appear to be functionally inconsequential^24,25^. As demonstrated in this report, the synaptic loss was observed by 3-weeks in the NOD mice. One can argue that once there is postsynaptic neural retraction from HCs, changes in SGN properties may be irrelevant.

Moreover, at early stages of hearing loss, reduction of synaptic contacts, together with decreased excitability of SGNs are likely to result in increased ABR peaks I and II delay and amplitude reduction as well as increased ABR thresholds. Degeneration of neuronal cell bodies is a slow process, with a substantial number of type II neurons loss preceding the death of type I neurons. Moreover, the finding may reflect the paucity of type II neurons, forming only ~5% of the neuron population in the spiral ganglion^36,37^. If the initial synaptic event serves as a catalyst for subsequent sensory cell degeneration, and it is reversible, then it is conceivable that intervention of the events leading to ARHL is feasible.

Mouse models of ARHL are known to be carriers of the alleles *Cdh23*^*ahl*^ and *ahl2*^12^. The *ahl* locus is located on the mouse chromosome 10 close to the *Cdh23* gene^12^. Sequence analyses of *Cdh23* gene in mouse models known to exhibit early-onset ARHL have shown single nucleotide substitution that leads to a frameshift mutation of exon 7^1,13^. Moreover, the cadherin 23 protein forms an essential component of hair bundle tip links and lateral links, and alterations of its structure resulting from mutations promote disarray of stereociliary bundles and HC loss^15,53^. The gene product of the other allele, *ahl2*, remains unknown. There have been suggestions that *ahl2* may represent a strial-associated protein, and could potentially be responsible for a decline in the endocochlear potential (EP) in the aging cochlear duct^14^. However, as demonstrated in this report and previous studies, the severity of hearing loss in the NOD mice was seen earlier than a reduction in the EP, which is a delayed occurrence (after six months old)^14^ and is unlikely to account for the product of *ahl2*. Instead, we predict that *ahl2* may encode a structural synaptic protein necessary for the maintenance of the IHC-spiral ganglion afferent synaptic functions. Alternatively, the possibility that *ahl2* may encode SGN-specific protein (e.g., ion channel) cannot be ruled out.

### Significant alterations in membrane input resistance and excitability in SGNs from aging NOD mice

Besides the loss of synaptic structures, an early and potentially important change observed in this report is a decline in membrane input resistance and excitability, resulting from increased outward K^+^ current magnitude. The normal ontogeny of a variety of excitable cells, including HCs, hippocampal neurons, and cells of the carotid body^54–56^ is accompanied by enhanced expression of specific K^+^ channel currents. Whereas the expression of these emerging outward currents may serve as a continuum of normal developmental refinement of neuronal firing^57^, the advent of K^+^ currents can transform an otherwise spontaneously active to quiescent cells, responding in a graded potential^56,58–60^. Additionally, previous studies have shown that senescent neurons have reduced membrane excitability^61^, attributed to the enhancement of transient and/or sustained K^+^ currents derived from K_v_3.1-related channels as well as Ca^2+^-activated K^+^ channels^62^. It remains unclear whether the latter can ensue from a rise in intracellular Ca^2+^, which is commonly associated with aging neurons^63^.

### Time course of altered SGN membrane properties, synaptopathy, and auditory phenotype

We observed significant alterations of membrane input resistance, resting membrane hyperpolarization, increased AP thresholds, and latency as early as in 2-week-old SGNs at the onset of hearing. Ultramicroscopic changes in SGN morphology were identified in 2-week SGNs. We assessed significant changes in pre- and post-synaptic mismatch by three weeks. These changes continued to worsen with time, and increased ABR thresholds were detected in 4-week-old NOD mice. We also identified changes in OHC numbers by 4-weeks in the diseased mice. Thus, while the study reveals relevant intrinsic changes in SGN membrane properties, neuronal and synaptic morphology as well as OHC loss, the results cannot be ascribed to a single sensory cell-type as the initiating target. Moreover, the data establish that early stages of hearing loss can originate from changes at multiple sensory cell-types.

### Future studies

Future studies are required to determine the identity of the K^+^ channel that is enhanced in the aging SGNs in the NOD model. Is the enhanced current products of the normal aging process, which is accelerated in NOD neurons, or is it a reflection of other unidentified aspects of the *ahl* allele products? There was a substantial, albeit statistically insignificant, increase in outward K^+^ currents in ICR control neurons from 2 to 12 weeks. Since the apparent developmentally regulated increase in the K^+^ current was small, it was not practical to evaluate the pharmacology or the identity of the channel current that undergo a normal progressive increase in ICR-control neurons. Thus, it is conceivable that the significant reduction in membrane input resistance, resulting from increased K^+^ current magnitude in SGNs in the NOD neurons, may originate from an entirely different etiology. These unknowns would require additional studies.

## Methods

### Assessment of inner ear functions

All experimental protocols were approved and carried out by the relevant guidelines and regulations in place at the University of Nevada Reno (NV), and Washington University, St Louis (MO). Additionally, the investigation was performed by the guidelines of the Institutional Animal Care and Use Committee of the University of Nevada, Reno. Auditory brainstem recordings (ABRs) and distortion product otoacoustic emission (DPOAE) recordings (assessment of outer hair cell functions) were performed on both the right and left ears of the NOD.CB17-Prkdc^scid^/J (NOD) and ICR/HaJ (ICR) mice at 4, 6, 8 and 12-week-old of age in a soundproof chamber maintained at 37 °C. Mice were anesthetized with ketamine (100 mg/kg) and xylazine (20 mg/kg) before recordings. Average ABR waveforms were plotted using MATLAB (MathWorks). Briefly, the ABR potentials were measured from needle electrodes positioned at the bottom of the tympanic bulla and the vertex of the head, with a ground electrode placed in the rear leg. The sound intensity level was raised in 10 dB steps from 10 to 100 dB sound pressure level (SPL), and the sound frequency was varied between 4 to 32 kHz. At each sound level, 260 responses were sampled and averaged. If an ABR response was not detected at 100 dB SPL, we arbitrarily set the threshold as 100 dB SPL for averaging purposes.

A probe tip microphone placed at the external auditory canal was used to measure distortion product otoacoustic emission (DPOAE). The sound stimuli for eliciting DPOAEs were two 1 second sine-wave tones of differing frequencies (F2 = 1.2 × F1). We varied the range of F2 from 4 to 32 kHz. The two tones were of equal intensities and stepped from 20 to 80 dB SPL in 2 dB increments. The amplitude of the cubic distortion product was measured at 2 × F1-F2. If a DPOAE was not detected at 80 dB SPL, we arbitrarily set the threshold as 80 dB SPL for averaging purposes.

### Isolation of spiral ganglion neurons (SGN)

Spiral ganglion neurons (SGNs) were isolated from the mouse inner ear using a combination of enzymatic and mechanical procedures^64^. Various ages of male and female (2–12-week old) ICR and NOD mice were sacrificed and the temporal bones were removed and incubated in a solution containing Minimum Essential Medium with HBSS (Invitrogen), 0.2 g/L kynurenic acid, 10 mM MgCl_2_, 2% fetal bovine serum (FBS; v/v), and glucose (6 g/L). The SGN tissue was then dissected and split into the apical, middle, and basal segments across the modiolar axis as previously described^65^. The tissues were digested separately in an enzyme mixture containing collagenase type I (1 mg/ml) and DNase (1 mg/ml) in a water bath with vibration rate 70/min at 37 °C for 15 mins. After a series of gentle trituration in centrifugation solution containing Minimum Essential Medium with HBSS, 0.2 g/l kynurenic acid, 10 mM MgCls, 10% PBS and glucose (6 g/l), the cell pellets were reconstituted in 500 μl of culture media (Neuralbasal-A, supplemented with 2% B27 (v/v), L-glutamine 0.5 mM, penicillin 100 U/ml; Invitrogen). The sample was filtered through a 40-μm cell strainer for additional cell culture and electrophysiological experiments. We cultured SGNs for ~48–72 h to allow the detachment of Schwann cells from neuronal membrane surfaces.

### Electrophysiology

For membrane voltage measurements in SGNs, the extracellular solution for most experiments contained the following (in mM): 145 NaCl, 6 KCl, 1 MgCl_2_, 0–2 CaCl_2_, 10 D-glucose, and 10 HEPES, at pH 7.3. For perforated patch experiments, the tips of the pipettes were filled with the internal solution containing the following (in mM): 150 KCl, 10 HEPES, and 10 D-glucose, at pH 7.3. The pipettes were front filled with the internal solution and backfilled with a similar solution containing 250 μg/ml amphotericin and 2 mM Ca^2+^. Thus, a switch from perforated patch to whole-cell mode results in rapid cell death. We examined the excitability of neurons using varying current magnitude and duration injection. Spontaneously active neurons were monitored without current injections.

For current measurements in SGNs, recordings were obtained using the whole-cell configuration of the patch-clamp technique. Signals were amplified using an Axopatch 200B (Molecular Devices) amplifier and filtered (bandpass 2–10 kHz). Data were digitized using an analog-to-digital converter (Digidata 1322; Molecular Devices). Patch electrodes were pulled from borosilicate glass capillaries with a Flaming/Brown microelectrode puller (P97, Sutter Instrument) and fire-polished to a final resistance of ~2–3 MΩ. The SGNs were then filled with the following internal solution in mM: 112 KCl, 2 MgCl_2_, 0.1 CaCl_2_, 5 MgATP, 0.5 Na_2_GTP, 1 EGTA or 10 BAPTA, 10 HEPES, pH 7.35 with KOH. The external solution consisted of (in mM): 130 NaCl or (5 NaCl and 125 NMGCl), 5 KCl, 1 MgCl_2_, 1 CaCl_2_, 10 D-glucose, and 10 HEPES, pH 7.4 with NaOH. The bath solution was constantly perfused at 2–3 ml/min. The liquid junction potentials were measured and adjusted as described^66^. All recordings were obtained at room temperature (20–22 °C).

### Data analysis

Data were analyzed using pCLAMP10.2 (Molecular Devices), Origin8.1 (MicroCal Software). All measurements were presented as mean ± SD, and Student’s test was used to determine statistical significance (*p* < 0.05). Analyses of voltage- and current-clamp data followed that described in detailed in previous reports from our laboratory^64,65,67^.

### Tissue isolation and whole-mount preparations of spiral ganglion neurons

We used NOD and aged-matched ICR mice as control animals. The animals were sacrificed, and their temporal bones removed and fixed in 4% paraformaldehyde in phosphate-buffered saline (PBS) for 2 hours on ice. The cochleae were fixed in 4% paraformaldehyde and then immersed in 10% EDTA in PBS for 3–5 days. The EDTA solution was changed daily. The tissues were rinsed three times (5 mins per rinse) in PBS. The decalcified cochlear bones were removed using fine forceps dissection, followed by removal of the lateral wall, stria vascularis (strial), Reissner’s membrane and tectorial membrane. The specimens were permeabilized with 0.5% Triton X-100 in PBS. Immunohistochemistry was performed by first blocking the samples with 10% goat serum (Jackson Immuno Research Laboratories, West Grove, PA) in PBST (PBS +0.5% Triton X-100) for 1 hour at room temperature and then incubated at 4 °C overnight in the following primary antibodies: mouse anti-Tuj1 (Covance), rabbit anti-Myo7a (Proteus Biosciences Inc.), mouse anti-C-terminal binding protein 2 (anti-CtBP2; BD Bioscience) and mouse anti- postsynaptic density protein 95 (anti-PSD95; Millipore). Specimens were washed three times with PBST and then incubated at room temperature for 2 hours with the following secondary antibodies: cyanine-5-conjugated (Cy5) goat anti-rabbit, cyanine-3-conjugated (Cy3) goat anti-mouse (Jackson ImmunoResearch Laboratories), Alexa Fluor 568 goat anti-mouse IgG1 and Alexa Fluor 488 goat anti-mouse IgG2a (Invitrogen). Samples were placed on a glass slide with a drop of fluorescent mounting medium (Electron Microscopy Sciences) and covered by glass coverslips. Images were acquired with a Zeiss LSM510 confocal microscope using either 40x or 63x/1.4 oil plan-apochromat objectives.

### Sample preparations for cryosection and immunolabeling

Decalcified cochleae were rinsed three times (30 minutes per rinse) in phosphate buffer saline (PBS). The cochleae were then immersed in 30% sucrose overnight for cryoprotection and embedded in OCT compound for cryo-sectioning at −20 °C. Cryosections with a thickness of 10 µm were washed three times before blocking for one hour at room temperature in blocking solution containing 10% goat serum in PBST (0.1% Triton X-100 in PBS). They were then incubated overnight at 4 °C in mouse anti-Tuj1 (Covance) and rabbit anti-peripherin (Millipore), both in an antibody solution of PBS +3% goat serum +0.1% Triton X-100. Sections were washed three times with PBST and then incubated for 2 hours at room temperature in Alexa Fluor 647 anti-mouse, Cy3 goat anti-rabbit, and FITC goat anti-rabbit (Jackson ImmunoResearch Laboratories, INC.). Specimens were washed three times, 5 minutes each, in PBS before mounting with fluorescent mounting medium (Electron Microscopy Sciences).

### Defining the pre- and post-synaptic overlap

All images for pre- and post-synaptic overlap analyses were captured using identical channel gains and confocal parameters. The objective is to determine the fraction of overlap of molecules CtBP2 (pre-synaptic structure) labeled with a red fluorophore and PSD95 molecules (post-synaptic structure) labeled with the green fluorophore. We define the overlap as conditional probabilities *P(R I G)* and *P(G I R)*. The first conditional probability is read as the probability of a red fluorophore occurring at a point *(x, y)* given that a green fluorophore is at *(x, y)*. The second conditional probability is read similarly with the terms red and green switched. The conditional probability P(R|G) is computed by

1 $$P(R|G)=N(R\sum G)/(N(G)$$

Where *N(R* Σ *G)* is the number of red and green pixels having the same (*x,y*) coordinates and *(N(G)* is the number of green pixels. Note that for any image the numerator is the same for both *P(R|G)* and *P(G|R)*.

### Determining the red and green regions

The fluorescence image of the CtBP2 label is arbitrarily assigned to the red image plane, and the image of the PSD 95 label is assigned to the green image plane. Before the conditional probabilities could be computed, we needed to expunge spurious objects from the images. We distinguish the nuclei from the nerve terminals by size. To do this, we first determine the median pixel intensity of the non-zero value pixels. A binary image was created where pixels whose value exceeded the median value were set to 1, and all others were set to zero. Scattered amongst the nuclei and nerve terminals were regions that had a sparse scattering of bright pixels. The scattered pixels could arise from poorly labeled objects, from objects outside of the focal plane, or simply noise. We eliminate these pixels while also identifying the nuclei using the ‘live-or-die’ algorithm^68^. The live-or-die algorithm eliminates pixels that have few neighbors and generates new pixels in regions that have a high density of pixels. Blobs (topologically path connected regions) having more than 300 pixels (an area about 17 × 17 pixels) were eliminated; preliminary measurements showed that this 300-cut-off (threshold intensity) value eliminated most of the nuclei while preserving the nerve terminals. Thus, using the ‘live-or-die algorithm, 300 pixel-cut-off, and visual inspection, we eliminated potential noise from the high-intensity synaptic signals. The nuclei did not appear in the red images, so filtering of the red images was simpler. The mean and standard deviation of the nonzero pixels were found. The binary image was created by setting all pixels whose value was lower than the mean +2 standard deviations to 0 and all other pixels to 1. The live-or-die algorithm was applied to this binary image.

### Sample preparations for histological evaluations

Anesthetized mice were trans-cardiac perfused with 0.1 M sodium cacodylate buffer followed by 4% paraformaldehyde and 2% glutaraldehyde or 1% osmium ferricyanide and 2% paraformaldehyde in the same buffer. The temporal bones were isolated, and perilymphatic perfusion was performed with the same fixative through the opened oval and round windows. The cochlea was immersed in the fixative for 1 hr (23 °C), decalcified in (120 mM EDTA, 2SS, pH 7.4, 23 °C) for 24–72-hr, dependent upon age, and then post-stained with 1% osmium tetroxide for 15–20 mins if needed. The cochleae were dehydrated through a series of graded ethanol and then infiltrated with resin (Embed 812, Electron Microscopic Sciences). The cochleae were transferred to a flat embedding mold. The plastic resin was polymerized (58 °C, 12 h) following which the cochleae were bisected through the modiolus and re-embedded with the mid-modiolar section facing the surface of the block. Thin cross-sections (70 nm) of the mid-modiolar cochlea were cut, mounted on copper grids and counterstained with UALC. The specimens were examined with a Hitachi H-7500 transmission electron microscope (TEM). Digitized images were acquired and archived with an Orca CCD camera with IC-PCI frame-grabber (Hamamatsu Photonics, Bridgewater, NJ) and AMT Advantage 12-hr software (Advanced microscopy techniques). Final images were prepared using Adobe Photoshop and Illustrator CS6.

## Data Availability

The datasets generated during and analyzed during the current study are available from the corresponding author on request.
